# Computerized Decision Support Systems Informing Community-Acquired Pneumonia Surveillance, France, 2017–2023

**DOI:** 10.3201/eid3011.240072

**Published:** 2024-11

**Authors:** Tristan Delory, Josselin Le Bel, Raphaëlle Métras, Caroline Guerrisi, Ilona E. Suhanda, Elisabeth Bouvet, Sylvie Lariven, Pauline Jeanmougin

**Affiliations:** Centre Hospitalier Annecy Genevois, Epagny Metz-Tessy, France (T. Delory); Antibioclic Steering Committee, Paris, France (T. Delory, J. Le Bel, E. Bouvet, S. Lariven, P. Jeanmougin); Sorbonne Université, INSERM, Institut Pierre Louis d'Épidémiologie et de Santé Publique, Paris (T. Delory, R. Metras, C. Guerrisi, I.E. Suhanda); Université Paris Cité, Paris (J. Le Bel); Université Paris Cité et Université Sorbonne Paris Nord, INSERM, Paris (J. Le Bel); Université de Nantes, Nantes, France (P. Jeanmougin)

**Keywords:** pneumonia, computerized decision support system, surveillance, bacteria, primary care, preparedness, community-acquired pneumonia, group A Streptococcus, Lyme disease, France, Mycoplasma pneumoniae, antimicrobial resistance

## Abstract

We show the value of real-time data generated by a computerized decision support system in primary care in strengthening pneumonia surveillance. The system showed a 66% (95% CI 64%–67%) increase in community-acquired pneumonia from 2018 to 2023 for the population of France, 1 month before a national alert was issued.

The COVID-19 pandemic has highlighted the importance of detecting novel or reemerging pathogens as they arise to enable the earliest possible response (*1*,*2*). The pandemic experience suggests that surveillance systems of routine health data collected at the primary healthcare level could rapidly identify emerging data patterns (signals) and inform future research to determine pandemic risk (*3*).

Since autumn 2023, health authorities in France have reported an increased rate of adults and children with pneumonia caused by *Mycoplasma pneumoniae*, including macrolide-resistant strains (*4*). *M. pneumoniae* circulates cyclically, with a higher rate in Europe and Asia every 3‒7 years (*5*). In Europe, serologic surveys have observed a decline in the detection of specific antibodies from 2020 to mid-2023 (*6*,*7*). Prospective serologic surveillance in 2023 showed increased incidence compared with previous years, consistent with a resurgence of *M. pneumoniae* (*8*). Diagnosis of atypical pneumonia in primary care is challenging; hospital-based serologic surveillance may misestimate the potential threat of the epidemic and is not scalable to primary care (*5*). Indeed, hospital-based surveillance often reports patients who have failed initial empiric therapy or have risk factors or complications.

The computerized decision support system (CDSS) Antibioclic (*9*) is designed for antimicrobial drug prescriptions for a panel of infectious diseases in primary care (*9*,*10*). Antibioclic could provide real-time information on the ecology and surveillance of community-acquired pathogens (*11*,*12*). The data collected by the CDSS are not linked to patients’ health records and do not allow patient identification (Appendix). Analyses of nonidentifiable data requests in Antibioclic do not require the approval of a research review board in France. Data collection and analysis follow European Union General Data Protection Regulation.

## The Study

We examined the pattern of requests for community-acquired pneumonia (CAP) within the Antibioclic system during November 11, 2017–January 7, 2024. We first calculated the weekly number of requests made for each type of pathology to the system; they were CAP, sore throat with positive group A *Streptococcus* (strep-A) test, and Lyme disease. We chose sore throat and Lyme disease for a baseline comparison to ensure that signal for CAP was not related to a change in the pattern of use in the CDSS. For sore throat with positive strep-A tests (1,595,867 requests) we observed a resurgence in children in late 2022 (*13*). Lyme disease (691,889 requests) is a vectorborne bacterial disease not known to be transmissible from person to person (*14*). 

We estimated the weekly incidence of requests made for each type of pathology per 1,000 overall requests (Appendix). The study period encompassed ≈27.7 million requests (21.4 million in adults and 6.3 million in children), of which 2,333,638 were for CAP (1,678,670 in adults and 567,849 in children), made mostly by primary care general practitioners (GPs) (92%, n = 46,762) (Appendix Table 1). Among requests performed in adults, 666,649 (39.7%) were for those >65 years of age, 417,094 (24.8%) involved other risk factors for severe CAP, and 189,304 (11.3%) were related to influenza-like illness (Appendix Table 2). We found that 1.49% (95% CI 1.46–1.52) of requests for CAP might be duplicated, defined as requests performed by a single user in <10 minutes. 

**Figure 1:**
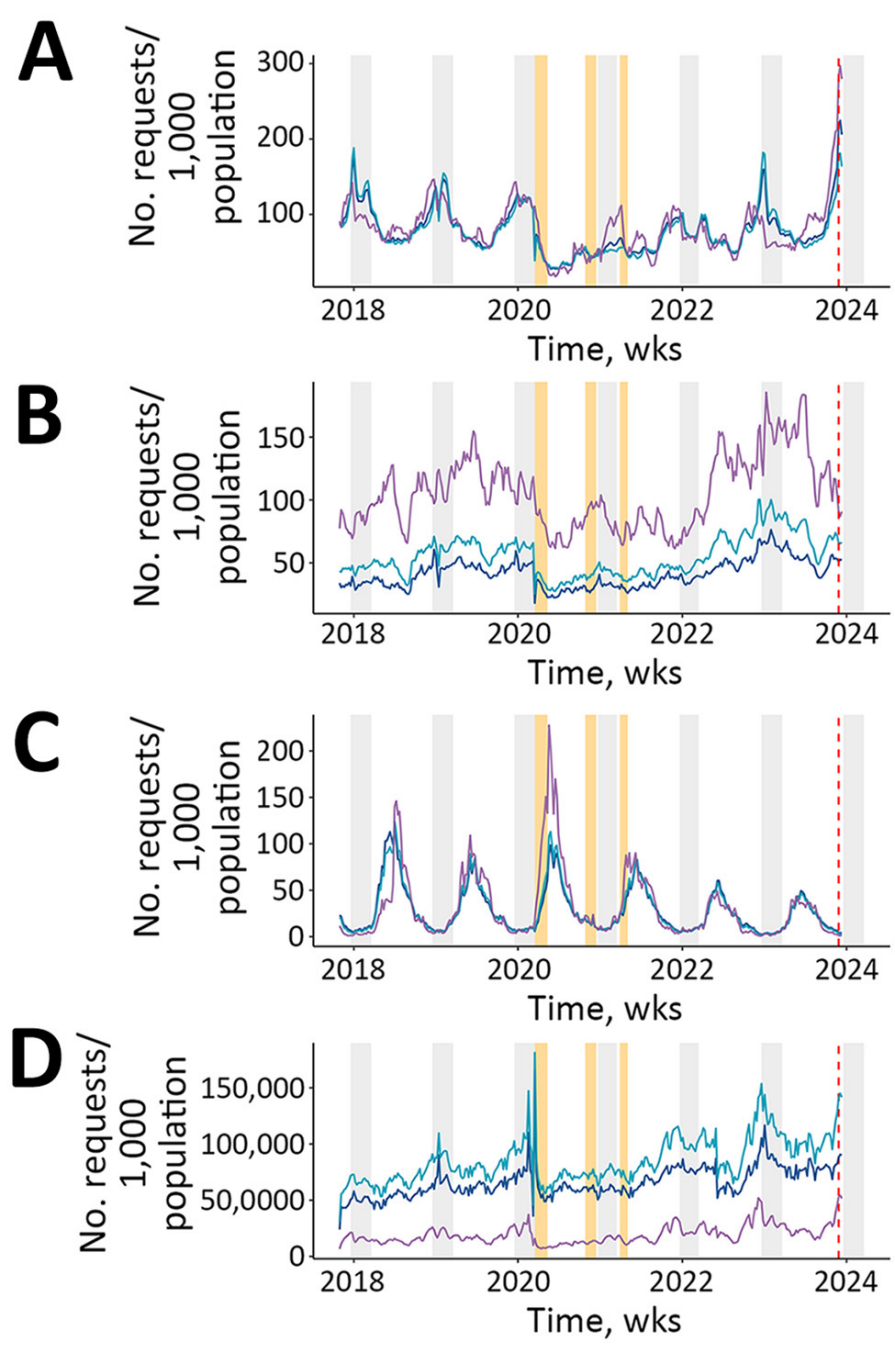
Temporal pattern of prescription data requests (per 1,000 population) within the Antibioclic computerized decision support system, France, December 2017–January 2024. A) Community-acquired pneumonia; (B) sore throat with positive group A *Streptococcus* test; C) Lyme disease; D) overall number of requests per week. Orange bars represent the 3 national lockdowns implemented in France during the COVID-19 pandemic. Light gray bars represent winter seasons. Purple lines represent evolution in children, light blue lines in adults, dark blue lines overall population. The dashed vertical red line represents the first national alert from the Ministry of Health associated with a possible outbreak of *Mycoplasma pneumoniae*, including macrolide-resistant strains.

We observed a seasonal pattern before the COVID-19 pandemic for both CAP and Lyme disease, winter peaks for CAP and summer peaks for Lyme disease; sore throat with positive strep-A test did not exhibit seasonal patterns (Figure 1). The COVID-19 pandemic affected the seasonal pattern of CAP. CDSS use was strongly reduced during the first lockdown; although its use recovered immediately after the release of the first restrictions, results showing seasonality of CAP did not resume until December 2022–March 2023. The seasonality of Lyme disease remains unchanged over the whole study period; peaks were as expected, in June 2022 and September 2023. We also observed a resurgence in streptococcal infections.

**Figure 2:**
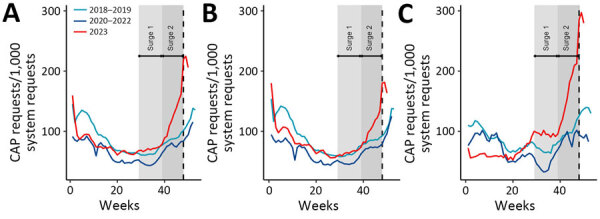
Rate of community-acquired pneumonia as indicated within the Antibioclic computerized decision support system, France, December 2017–January 2024. A) Overall population; (B) adults; (C) children. Light blue line indicates average number of system requests in 2018–2019 (pre‒COVID-19 pandemic); dark blue line indicates average number of system requests in 2020–2022 (during pandemic); red line indicates average number of system requests in 2023. Light gray area indicates a surge in 2023 starting in epidemiologic week 29 and dark gray indicates surge in 2023 starting in week 39; we noted that surges began earlier in children than adults. The dashed vertical line represents the first national alert from the Ministry of Health associated with a possible outbreak of *Mycoplasma pneumoniae.*

To compare seasonal patterns, we calculated rates by quarters and years for the whole study period (2018‒2023) in the whole population and in adults and children. Then, we estimated relative risks (RRs) by comparing the quarterly rate for year 2023 to the same quarter for year 2018. The peak rate in winter 2022‒2023 and 2017‒2018 was ≈175 CAP/1,000 requests. However, from November 2023 onward, we observe an increase in incidence of CAP requests compared with previous years. The quarterly evolution between the reference year, 2018, and 2023 showed that both children and adults faced a resurgence of CAP during the 4th quarter but that the increase was higher for children: RR was 1.66 (95% CI 1.64–1.67) overall, 1.48 (95% CI 1.46–1.49) in adults, and 1.87 (95% CI 1.84–1.89) in children (Table). The resurgence also started earlier in children than in adults, which we observed in epidemiologic week 29 of 2023 (from 80 CAP/1,000 requests to 100 CAP/1,000 requests) and for weeks 39–52 of 2023 (Figure 2). In adults, the resurgence started during the 4th quarter, in week 40 of 2023. 

**Table:** Prescription data requests within the Antibioclic computerized decision support system for community-acquired pneumonia, France, 2018–2023*

Quarter	No. requests/1,000 population						2023 vs. 2018 relative risk (95% CI)
	2018	2019	2020	2021	2022	2023	
Overall							
1	126.1	117.9	102.9	59.0	78.8	95.9	0.76 (0.75–0.77)
2	74.2	73.6	35.4	51.4	72.0	67.5	0.91 (0.90–0.92)
3	68.0	62.1	37.7	53.7	57.9	69.9	1.03 (1.02–1.04)
4	99.9	100.7	47.9	89.7	104.4	165.4	1.66 (1.64–1.67)
Adults							
1	135.5	120	99.4	51.9	80.2	107.9	0.80 (0.79–0.80)
2	73.9	73.4	35.9	47.4	74.1	68.9	0.93 (0.92–0.94)
3	65.7	60.2	37.8	53.4	57.7	63.8	0.97 (0.96–0.98)
4	93.4	95.5	48.0	86.3	107.0	138.0	1.48 (1.46–1.49)
Children							
1	94.6	110.8	116.5	84.8	74.0	60.2	0.64 (0.62–0.65)
2	75.1	74.5	31.6	67.8	65.3	62.8	0.84 (0.82–0.85)
3	77.7	70.3	37.1	55.1	58.7	94.0	1.21 (1.18–1.24)
4	119.9	117.0	47.2	99.1	98.6	223.6	1.87 (1.84–1.89)

*The rate of community-acquired pneumonia sharply decreased in all groups after introduction of nonpharmaceutical interventions for the COVID-19 pandemic (from the second quarter of 2020).

The Ministry of Health in France issued a national alert 7 weeks (during week 47) after the start of the second surge involving both children and adults, 4 weeks after the rate of CAP rose by 25% in our system compared with prepandemic years. The peak during 2024 epidemiologic week 1, at 253 CAP/1,000 requests overall, corresponds to a 46% increase compared with the same week in winter 2017–2018, stratified as 232 CAP/1,000 adults (23% higher than 2017–2018) and 310 CAP/1,000 children (269% higher than 2017–2018).

Finally, we estimated the expected numbers of CAP in 2023 in the absence of a resurgence. We trained a Poisson model with the 2018–2019 data (Appendix), projected for 2023 and compared those estimates to the observed 2023 data to compute excess CAP requests. We estimate an excess 17,876 requests (14.4% increase) for CAP (9,205 [9.9% increase] in adults, 8,671 [27.9% increase] in children) in 2023 compared with 2018–2019.

## Conclusions

By analyzing the requests of CAP and 2 other control pathologies, we showed that Antibioclic data are successful in detecting early emergence of atypical CAP, observed elsewhere in Europe (*8*). This dataset, which covers many pathologies in primary care (n = 36), could be leveraged to monitor localized or national-level outbreaks and contribute to the assessment of emerging threats. Primary healthcare CDSSs that provide real-world and real-time data may effectively support pandemic pathogen intelligence by detecting or confirming signals of disease outbreaks. Those results can strengthen local surveillance or inform global surveillance centers. Our system does not identify specific pathogens involved in CAP and is not integrated into primary-care electronic health records (*9*). We plan to refine our estimates using field data. 

**Figure 3:**
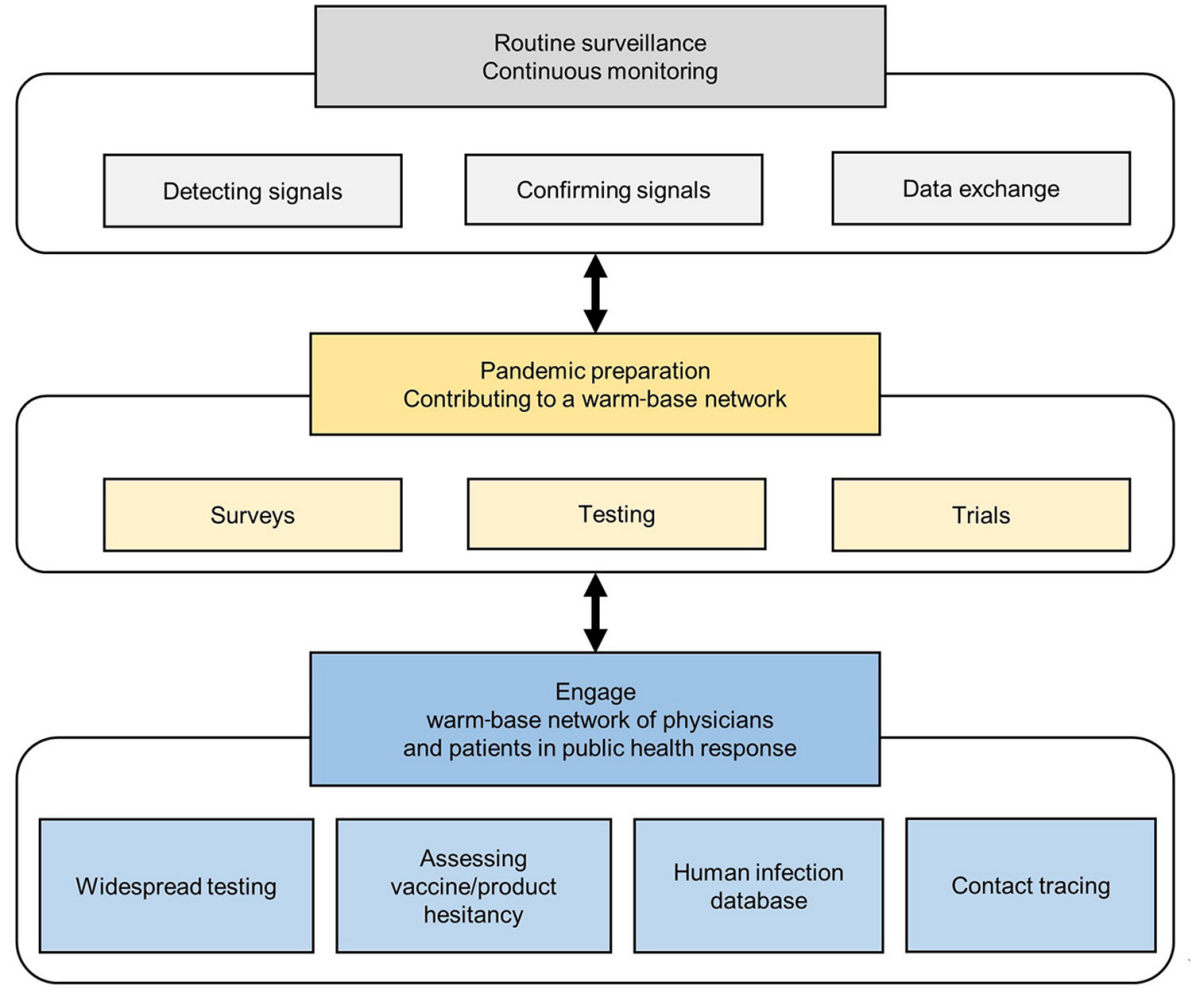
Potential contribution from primary healthcare computerized decision support system to global networks for pandemic preparedness by routine surveillance and continuous monitoring and a ready (warm-base) network of primary health care physicians already using the system that can be engaged for public health response in case of pandemic.

In France, ≈57% of 80,000 GPs use the CDSS; users are more likely to be younger than average (39 vs. 51 years of age), and more likely to be female (63.0% vs. 46.9%) than the whole population of GPs (*15*). Yet, CDSS was successful in confirming reemergence of CAP. Wide use of CDSS in primary care enabled creation and maintenance of a warm-base network of primary care physicians ready to engage against a pandemic (Figure 3). Those physicians could carry out widespread testing of the general population, improve contact tracing, and contribute to human infection databases. They could also be involved in surveys to better understand behaviors that lead to hesitancy toward new vaccines or products and use the knowledge gained to develop strategies to increase uptake before products are introduced. 

Effective end-to-end communication between stakeholders, CDSS administrators, and users enables health authorities to maximize public information and health response. Between emerging or reemerging signals, the network can prepare by involving its users and their patients in surveys, tests, and trials.

Appendix Additional information about the use of computerized decision support systems to inform community-acquired pneumonia surveillance, France, 2017–2023.
