# Supplementary material for: Computerized Decision Support Systems Informing Community-Acquired Pneumonia Surveillance, France, 2017–2023
Source: Emerg Infect Dis. 2024 Nov;30(11):2404–8. doi: 10.3201/eid3011.240072 (PMC11521177; doi:10.3201/eid3011.240072)
Supplement: Appendix — Additional information about the use of computerized decision support systems to inform community-acquired pneumonia surveillance, France, 2017–2023. [file 24-0072-Techapp-s1.pdf]

Article DOI: http://doi.org/10.3201/eid3011.240072

EID cannot ensure accessibility for supplementary materials supplied by authors. Readers who have difficulty accessing supplementary content should contact the authors for assistance.

Computerized Decision Support Systems Informing Community-Acquired Pneumonia Surveillance, France, 2017–2023

Appendix

Computation of Incidence

We estimated the weekly incidence of requests $I_{i,j}$ made per 1000 overall requests for each type of pathology, as follow:

$$I_{i,j} = \frac{1000 \, * \, N_{i,j}}{T_j}$$

With $N_{i,j}$ being the number of requests of pathology i in week j, and T_j the overall total number of requests for that week.

Estimation of Excess of Community-Acquired Pneumoniae

To estimate the excess number of community-acquired pneumoniae in 2023, we fitted a Poisson regression of the monthly counts of CAP with the overall number of requests as an offset, adjusted on age group (adult or children), with a random intercept for month and a sinusoidal component to account for seasonality. We trained the model (Supplemental Materials) with the 2018–2019 data, projected for 2023 and compared these to the observed 2023 data to compute the excess CAP requests.

Data Collected When Physicians Are Performing Requests

Data collected during requests performed to the CDSS are filled by physicians and does not the identification of patients. A summary of collected information is published in the article we issued in the *Journal of Antimicrobial Chemotherapy* in 2020 by Delory et al (9). We are not accessing health records. The data collection process ensures that we cannot identify patients.

The data we are collecting are considered anonymized and not categorized as pseudo-anonymized health data. According to French regulation (https://www.cnil.fr/fr/quest-ce-ce-quune-donnee-de-sante), we are not enrolling patients and we are analyzing requests. Thereby, we have to follow the French regulation aligned with the EU GDPR (https://gdpr-info.eu/). Our confidentiality policy details all the data that we are collecting and how we can analyze them: https://antibioclic.com/p/politique-de-confidentialite

We are routinely collecting data used for describing a clinical case:

- Anatomic domain related to the pathology/infectious disease.
- Name of the pathology/infectious disease.
- Age group: adult or children. Age is not collected as a numerical value.
- Physician postal code, which allow spatial analyses aggregated at the physician's level.

We also collect specific conditions that might impact the choice of antibiotic therapy and drug–drug interactions:

- Breastfeeding (in adults)
- Pregnancy (in adults)
- Chronic kidney failure (in adults and children)

Specifically, for CAP in adults, we additionally collect data used to rank CAP severity in the perspective of a primary health care physician:

- Age >65 years or ≤65 years;
- Other risk factors than age >65 years (chronic cardiac failure, or stroke, or chronic kidney disease, or chronic liver failure, or COPD, or immunocompromised (steroid intake, HIV, ongoing chemotherapy…), or homozygote sickle-cell disease, or history of bacterial CAP, or hospitalization in the previous 12 months, or living in a nursing home): yes/no;
- Influenza-like illness context: yes/no

Appendix Table 1. Characteristics of CDSS users from primary care as of January 1, 2023

CDSS users' characteristics	N = 61,792<br>N (%) or median [InterQuartileRange]
Sex, *female*	38,961 (63.0%)
Age	39 y [34– 51]
Medical specialty – top 5	
General practitioners	46,762 (92.0%)
Paediatrician	663 (1.3%)
Gynecologists	556 (1.1%)
Emergency physicians	297 (0.6%)
Geriatrics	293 (0.6%)
Mentoring students, *yes*	15,707 (25%)

Appendix Table 2. Details of requests for CAP in adults

	Requests for CAP in adults<br>N = 1,678,670	
Request's characteristics	N	% (95CI)
Age >65 y	666,649	39.7% (39.6% to 39.8%)
Pregnancy	49,836	2.9% (2.9% to 3.0%)
Breastfeeding	15,512	0.9% (0.9% to 0.9%)
Chronic kidney disease	58,160	3.5% (3.4% to 3.5%)
Other risk factors for severe CAP than age >65 y*:	417,094	24.8% (24.8% to 24.9%)
Age >65 y with ≥1 additional risk factor for severe CAP	196,608	11.7% (11.6% to 11.8%)
Influenza-like illness context	189,304	11.3% (11.2% to 11.3%)

* Risk factors for severe CAP among the following: chronic cardiac failure, stroke, chronic kidney disease, chronic liver failure, COPD, immunocompromised (steroid intake, HIV, ongoing chemotherapy…), homozygote sickle-cell disease, history of bacterial CAP, hospitalization in the previous 12 months.
